# A method for generating synthetic longitudinal health data

**DOI:** 10.1186/s12874-023-01869-w

**Published:** 2023-03-23

**Authors:** Lucy Mosquera, Khaled El Emam, Lei Ding, Vishal Sharma, Xue Hua Zhang, Samer El Kababji, Chris Carvalho, Brian Hamilton, Dan Palfrey, Linglong Kong, Bei Jiang, Dean T. Eurich

**Affiliations:** 1 Replica Analytics Ltd, Ottawa, ON Canada; 2 Children’s Hospital of Eastern Ontario Research Institute, 401 Smyth Road, Ottawa, ON K1J 8L1 Canada; 3 School of Epidemiology and Public Health, University of Ottawa, Ottawa, ON Canada; 4 Department of Mathematical and Statistical Sciences, University of Alberta, Edmonton, AB Canada; 5 School of Public Health, University of Alberta, Edmonton, AB Canada; 6 Health Cities, Edmonton, AB Canada; 7 B W Hamilton Consulting Inc., Edmonton, AB Canada; 8 Institute of Health Economics, Edmonton, Alberta Canada

**Keywords:** Synthetic data, Administrative health data, Data privacy, Data sharing

## Abstract

Getting access to administrative health data for research purposes is a difficult and time-consuming process due to increasingly demanding privacy regulations. An alternative method for sharing administrative health data would be to share synthetic datasets where the records do not correspond to real individuals, but the patterns and relationships seen in the data are reproduced. This paper assesses the feasibility of generating synthetic administrative health data using a recurrent deep learning model. Our data comes from 120,000 individuals from Alberta Health’s administrative health database. We assess how similar our synthetic data is to the real data using utility assessments that assess the structure and general patterns in the data as well as by recreating a specific analysis in the real data commonly applied to this type of administrative health data. We also assess the privacy risks associated with the use of this synthetic dataset. Generic utility assessments that used Hellinger distance to quantify the difference in distributions between real and synthetic datasets for event types (0.027), attributes (mean 0.0417), Markov transition matrices (order 1 mean absolute difference: 0.0896, sd: 0.159; order 2: mean Hellinger distance 0.2195, sd: 0.2724), the Hellinger distance between the joint distributions was 0.352, and the similarity of random cohorts generated from real and synthetic data had a mean Hellinger distance of 0.3 and mean Euclidean distance of 0.064, indicating small differences between the distributions in the real data and the synthetic data. By applying a realistic analysis to both real and synthetic datasets, Cox regression hazard ratios achieved a mean confidence interval overlap of 68% for adjusted hazard ratios among 5 key outcomes of interest, indicating synthetic data produces similar analytic results to real data. The privacy assessment concluded that the attribution disclosure risk associated with this synthetic dataset was substantially less than the typical 0.09 acceptable risk threshold. Based on these metrics our results show that our synthetic data is suitably similar to the real data and could be shared for research purposes thereby alleviating concerns associated with the sharing of real data in some circumstances.

**Supplementary Information:**

The online version contains supplementary material available at 10.1186/s12874-023-01869-w.

## Background

It is often difficult for analysts and researchers to get access to high quality individual-level health data for research purposes. For example, despite funder and journal expectations for authors to share their data [1–3], an analysis of the success rates of getting individual-level data for research projects from authors found that the percentage of the time these efforts were successful varied significantly and was generally low at 58% [4], 46% [5], 14% [6], and 0% [7]. Some researchers note that getting access to datasets from authors can take from 4 months to 4 years [7]. Data access through independent data repositories can also take months to complete [8, 9].

Concerns about patient privacy, coupled with increasingly strict privacy regulations, have contributed to the challenges noted above. For instance, privacy concerns by patients and regulators have acted as a barrier to sharing of health data [10, 11]. A recent review of health data infrastructure in Canada concluded that (mis)interpretations of privacy laws and a general “privacy chill” incentivizes risk-averse behavior among data custodians, stifling data access and research [12]. An analysis of data sharing practices for studies funded by CIHR found nontrivial gaps in data availability [13]. There are a number of approaches that are available to address these concerns: consent, anonymization, and data synthesis.

Patient (re-)consent is one legal basis for making data available to researchers for secondary purposes. However, it is often impractical to get retroactive consent under many circumstances and there is significant evidence of consent bias [14].

Anonymization is one approach to making clinical and administrative data available for secondary analysis. However, recently there have been repeated claims of successful re-identification attacks on anonymized data [15–21], eroding public and regulators’ trust in this approach [21–31].

Data synthesis is a more recent approach for creating non-identifiable health information that can be shared for secondary analysis by researchers [32, 33]. Researchers have noted that synthetic data does not have an elevated identity disclosure (privacy) risk [34–41], and recent empirical evaluations have demonstrated low disclosure risk [42]. Synthetic data generation has the potential to unlock historically siloed and difficult to access data sets for secondary analysis, including research.

There are synthetic health datasets that are currently available to a broad research community such as: the NIH National COVID Cohort Collaborative (N3C) [43], the CMS Data Entrepreneur’s Synthetic Public Use files [44], synthetic cardiovascular and COVID-19 datasets available from the CPRD in the UK [45, 46], A&E data from NHS England [47], cancer data from Public Health England [48], a synthetic dataset from the Dutch cancer registry [49], synthetic variants of the French public health system claims and hospital dataset (SNDS) [50], and South Korean data from the Health Insurance Review and Assessment service (the national health insurer) [51].

There are multiple methods that have been developed for the generation of cross-sectional synthetic health data [52–59]. The synthesis of longitudinal data is more challenging because patients can have long sequences of events that need to be incorporated into the generative models. Longitudinal data captures events and transactions over time, such as those in electronic medical records, insurance claims datasets, and prescription records. As we summarize below, published methods thus far are not suitable for the synthesis of realistic longitudinal data because many of them would have only worked with curated data where the messiness of real-world data has been taken out.

In this article we present a recurrent neural network (RNN) model for the generation of synthetic longitudinal health data. The model was empirically tested on Alberta’s administrative health records. Individuals were selected for this cohort if they received a prescription for an opioid during the 7-year study window. Data available for this cohort of patients included demographic information, laboratory tests, prescription history, emergency department visits, hospitalizations, and death. The synthesized data utility was evaluated using generic metrics to compare the real data with the synthetic data, and a traditional time-to-event analyses on opioid use was performed on both datasets and the results compared. This type of analysis is the cornerstone of most health services research. The privacy risk associated with the synthetic dataset was assessed using an attribution disclosure risk assessment on synthetic data [42].

## Methods

In this section we describe the requirements for a generative model that captures the patterns in complex longitudinal clinical datasets, and a RNN architecture to meet those requirements. We also describe how we evaluate the utility and privacy risks of the generated datasets.

The utility of the generated data can be evaluated using two approaches [60]: general purpose utility metrics and a workload aware evaluation. The former approach evaluates the extent to which the characteristics and structure of the synthetic data are similar to characteristics of the real data, and the latter compares the model results and conclusions of a substantive analysis on opioid use utilizing the synthetic and real datasets. We performed both types of utility assessment.

### Requirements for synthesizing longitudinal health data

We first present a series of requirements for the synthesis of longitudinal health data. This allows us to be precise in evaluating previous work and for setting express target criteria for our generative model. These requirements are intended to capture: (a) the characteristics of real longitudinal datasets that have received minimal curation to ensure that the synthesized datasets are realistic and that the generative models will work with real health data, and (b) the characteristics of the generative models themselves to ensure that they are scalable and generalizable. Our requirements are as follows:

1. The original dataset that is synthesized is a combination of:
  - Longitudinal data (i.e. multiple events over time from the same patient), and
  - Cross-sectional data (i.e., measures that are fixed and are not repeated such as demographic information).
2. The length of the longitudinal sequence varies across patients in the original datasets. Patients with acute conditions may have very few events, whereas complex patients with chronic conditions may have a very large number of events.
3. The original datasets are heterogeneous with a combination of:
  - Categorical or discrete features
  - Continuous features
  - Categorical variables with high cardinality (e.g., diagnosis codes and procedure codes)
4. Outliers and rare events should be retained in the original dataset since real data will have such events in them.
5. The data may have many missing values, leading to sparse datasets (i.e., missing data are not removed from the original datasets that are synthesized).
6. The generative model can take into account the previous information about the patients in the sequence.
7. The generative model should be developed based on existing data rather than requiring manual intervention by clinicians to seed it or correct it.

Our objective was to construct a generative model that would meet these requirements.

### Previous approaches

Multiple methods have been proposed in the literature for synthesizing longitudinal health data, each with their own strengths and limitations. These are summarized in Table 1. None of them meet all of our requirements, making the case for additional research and generative model architectures to meet the requirements above.

**Table 1:** Literature review of key characteristics of previous works for generating longitudinal synthetic health data

Title	Data Structure			Variable Types					Model Types	
	Cross-sectional R.1a)	Longitudinal (R.1b)	Variable length sequences (R.2)	Categorical (R.3a)	Continuous (R.3b)	Categories with high cardinality (R.3c)	Outliers removed (R.4)	Missing values present in data (R.5)	Consider all the previous information (R.6)	Model informed by clinicians (R.7)
Variational Autoencoder Modular Bayesian Networks (VAMBN) for Simulation of Heterogeneous Clinical Study Data [61]	No	Yes	Fixed	Yes	Yes	Yes	N/D	Yes	Yes	No
Machine learning for comprehensive forecasting of Alzheimer’s Disease progression [62]	No	Yes	Varied	Yes	Yes	No	N/D	Yes	No	No
Design and Validation of a Data Simulation Model for Longitudinal Healthcare Data [63]	No	Yes	Varied	Yes	No	Yes	N/D	No	Yes	No
Privacy-Preserving Generative Deep Neural Networks Support Clinical Data Sharing [64]	No	Yes	Fixed	No	Yes	No	Yes	No	Yes	No
Analyzing Medical Research Results Based on Synthetic Data and Their Relation to Real Data Results: Systematic Comparison From Five Observational Studies [65]	Yes	No	N/A	Yes	Yes	No	N/D	Yes	N/A	No
Synthetic Event Time Series Health Data Generation [66]	Yes	Yes	Fixed	Yes	Yes	No	Yes	No	Yes	No
Data-driven approach for creating synthetic electronic medical records [67]	No	Yes	Varied	Yes	Yes	Yes	N/D	N/D	Yes	No
Synthea: An approach, method, and software mechanism for generating synthetic patients and the synthetic electronic health care record [68]	Yes	Yes	Varied	Yes	Yes	Yes	N/D	No	Yes	Yes
Real-valued (medical) time series generation with recurrent conditional GANS [69]	No	Yes	Fixed	No	Yes	N/A	Yes	No	Yes	No
Generating Multi-label Discrete Patient Records using Generative Adversarial Networks [70]	Yes	No	N/A	Yes	No	No	N/D	Yes	No	No
Data Synthesis based on Generative Adversarial Networks [35]	Yes	Yes	Fixed	Yes	Yes	Yes	N/D	N/D	Yes	No
Generation and Evaluation of Privacy Preserving Synthetic Health Data [71]	Yes	No	N/A	Yes	Yes	Yes	No	No	No	No
Generation of Heterogeneous Synthetic Electronic Health Records using GANs [72]	Yes	No	N/A	Yes	Yes	Yes	Yes	N/D	No	No
Generating Electronic Health Records with Multiple Data Types and Constraints [73]	Yes	No	N/A	Yes	Yes	Yes	Yes	N/D	No	No
Ensuring electronic medical record simulation through better training, modeling, and evaluation [74]	Yes	No	N/A	Yes	No	Yes	Yes	N/D	No	No
Generative Adversarial Networks for Electronic Health Records: A Framework for Exploring and Evaluating Methods for Predicting Drug-Induced Laboratory Test Trajectories [75]	No	Yes	Fixed	No	Yes	N/A	Yes	No	Yes	No
Synthesizing electronic health records using improved generative adversarial networks [76]	Yes	No	N/A	Yes	No	No	Yes	N/D	Yes	No
Generating Fake Data Using GANs for Anonymizing Healthcare Data [77]	Yes	Yes	Fixed	Yes	Yes	No	Yes	N/D	No	No
CorGAN: Correlation-Capturing Convolutional Generative Adversarial Networks for Generating Synthetic Healthcare Records [78]	Yes	No	N/A	Yes	Yes	No	N/D	N/D	N/A	No
Generation and evaluation of synthetic patient data [79]	Yes	No	N/A	Yes	Yes	No	No	N/D	N/A	No
Generating and Evaluating Synthetic UK Primary Care Data: Preserving Data Utility & Patient Privacy [80]	Yes	No	N/A	Yes	Yes	No	No	N/D	N/A	No
SMOOTH-GAN: Towards Sharp and Smooth Synthetic EHR Data Generation [81]	Yes	No	N/A	Yes	Yes	No	Yes	N/D	N/A	No
Continuous Patient-Centric Sequence Generation via Sequentially Coupled Adversarial Learning [82]	No	Yes	Varied	No	Yes	N/A	Yes	No	Yes	No
Medical Time-Series Data Generation using Generative Adversarial Networks [83]	No	Yes	Varied	Yes	Yes	No	N/D	N/D	No	No

*N/A* refers to not applicable while *N/D* refers to not described

### Data characteristics

We used a cohort of patients previously derived and published to evaluate trends in opioid use in the province of Alberta, Canada [84]. The following administrative databases from Alberta Health from 2012 to 2018 were linked by the encrypted personal health number (PHN).

1. The Provincial Registry and Vital Statistics database for patient demographics and mortality. We used the age, sex, vital statistics, and date of last follow-up. An additional covariate was derived, the Elixhauser comorbidity score, based on physician, emergency department or hospitalization ICD-9/10 codes.
2. Dispensation records for pharmaceuticals from the Alberta Netcare Pharmaceutical Information Network (PIN). We restricted the data to only dispensations of either one of two commonly dispensed opioids of interest in our data (morphine and oxycodone) and dispensations of antidepressant medications since these were the focus of the time-to-event analysis.
3. The Ambulatory Care Classification System which provides data on all services while under the care of the Emergency Department. This included date of the visit, primary diagnostic codes, and resource intensity weight. The resource intensity weight is a measure used in the province to determine the amount of resources used during the visit. The primary diagnostic code we included as an ICD-10 code.
4. Discharge Abstract Database which provides similar data to Ambulatory Care but pertaining to inpatient hospital admissions. Information on hospitalizations was restricted to the date of admission, primary diagnostic code, and the resource intensity weight. 
5. Provincial laboratory data which includes all outpatient laboratory tests in the province. We only considered results for 3 common labs conducted in the province (ALT, eGFR, HCT) and the associated date of testing.

**Fig. 1:**
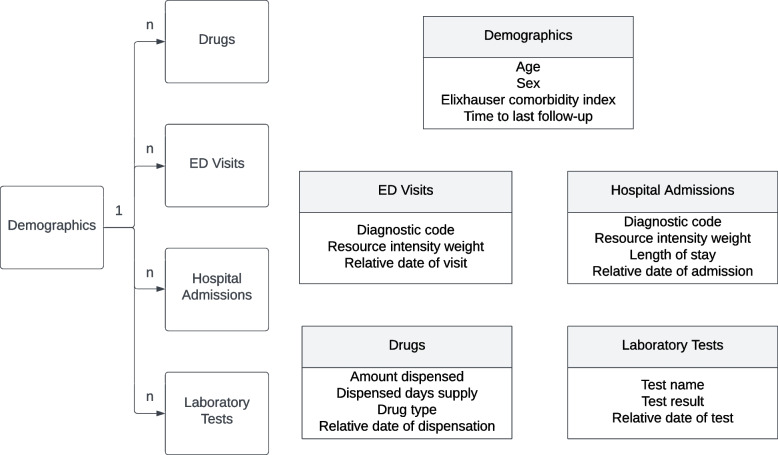
Representation of the dataset. Note that the demographics information contains a single observation per individual, where each individual is identified using a personal health number (PHN). This PHN links the demographics table to all other tables in the dataset, where all other tables may have multiple observations per individual

The structure of the data is illustrated in Fig. 1. There is a demographic table with basic characteristics of patients, and a set of transactional tables with a one-to-many relationship between the demographic table and the transactional tables. Therefore, each patient may have multiple events occurring over time. Using the PHN, observations for a single individual from multiple transactional tables may be linked together. Each observation in the transactional tables includes the date of the event relative to the start of the study period. This means that a group of observations from the same individual can be sorted according to the relative date, yielding a chronological order of an individual’s interactions with the health system.

Each event, whether it is a visit or a lab test, has a different set of attributes. Therefore, the event characteristics are a function of the event type. For example, a hospitalization event will record the relative date of the hospitalization, the length of stay, diagnostic code, and resource intensity weight. Additionally, all event types include an attribute to describe the timing of the event. In this work we model time using sojourn time, or time in days since the last event for that individual.

The basic patient characteristics and event characteristics are heterogeneous in data type. This means that some will be categorical variables, some will be continuous, some binary, and some discrete ordered variables. For example, age is a continuous patient characteristic while diagnostic code associated with a emergency department visit is a categorical event characteristic.

Table 2 provides the exact dimensionality of the original datasets. A random subset of 100,000 patients from a population of 300,000 subjects who received a dispensation for morphine or oxycodone between Jan 1, 2012 and Dec 31, 2018, 18 years of age and over were used to train our generative model and included in our analyses. For these patients, we truncated the events at the 95th percentile, which means that the maximum number of events that an individual can have was 1000.

**Table 2:** Dimensionality of the original data tables for the 100,000 individuals used for training

Table Name	Number of Rows	Number of Columns
Age_sex_comorbidity	100,000	4
Drug_data	9,975,950	7
ED_visits	1,748,083	5
Hosp_admit	84,669	5
Labs	2,199,574	3
Reg_file	100,000	2
Vital_stats	4200	6

Details of the dataset preparation for the modeling are provided in Additional file 1: Appendix 1: Data Pre-processing.

### Generative model description

To synthesize complex longitudinal health data, we use an RNN. RNNs model input sequences using a memory representation which is aimed to capture temporal dependencies. Vanilla RNNs, however, suffer from the problem of vanishing gradients [85] and thus, have difficulty capturing long-term dependencies. Long short-term memory units (LSTM) [86] and the gated recurrent unit (GRU) [87] were conceived to overcome this limitation. This work implements LSTM to model and synthesize observations over time. The generated data was then evaluated in terms of data utility. This generative model was implemented in python version 3.8 using Pytorch version 1.7.

#### Model structure

**Fig. 2:**
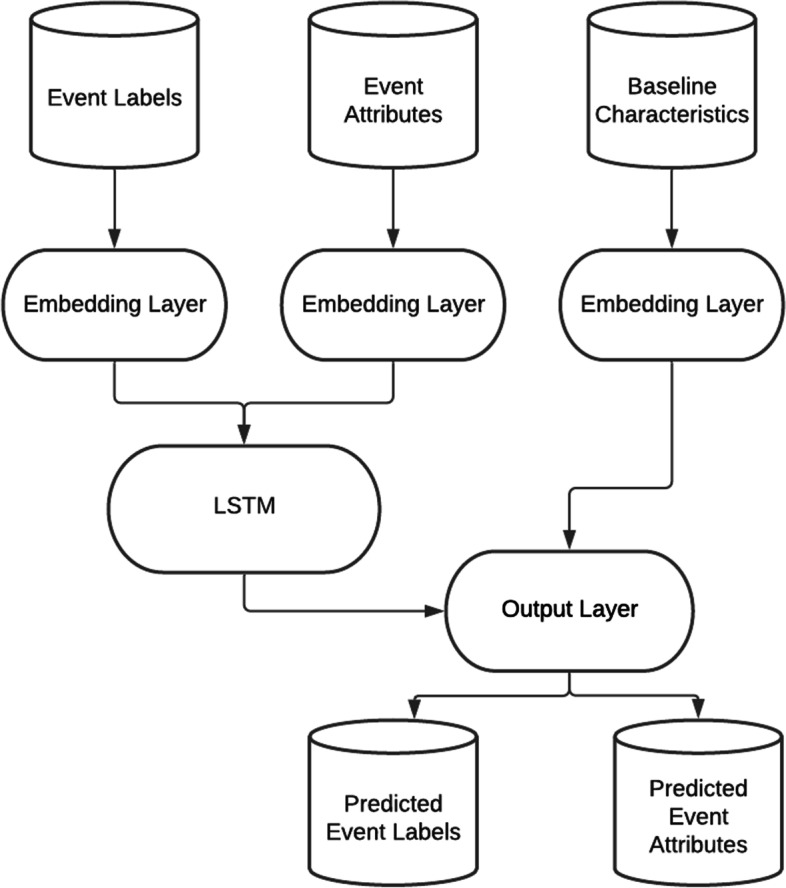
Diagram of the overall RNN architecture

Our generative model was a form of conditional LSTM where the final predicted outputs are conditional on the baseline characteristics. The model architecture, including which datasets are provided as inputs vs predicted as outputs is described in Fig. 2. The input data corresponds to *n* individuals at *t* − 1 time points (e.g., the set *Tϵ* {1, 2, 3, .. *t* − 1}) for event labels (yielding an array of dimensions [*n*, *t* − 1]) and event attributes (yielding an array of dimensions [*n*, *t* − 1, *A*] where *A* is the number of attributes) as well as the *B* baseline characteristics for each individual. The output consists of predictions corresponding to *n* individuals at *t* − 1 time points (e.g., the set *Tϵ* {2, 3, 4, .. *t*}) for the event labels and event attributes. These predictions are used during training to calculate the model loss, or during data generation as the subsequent synthetic events.

This model consists of 3 components: embedding layers, an LSTM, and output layers.

The embedding layers are used to map single integer encoded categorical features to a series of continuous features. The benefit of this embedding is that the transformation to map the discrete features to the set of continuous features is altered and improved throughout training. This allows for a continuous space representation of the categorical features that picks up similarity between related categories. Embedding occurs independently for each of the baseline characteristics (age, sex, comorbidity index), the event labels, and the event attributes.

The LSTM estimates a representation of the hidden state given the prior event labels and attributes. The embedded event attributes and the embedded event labels are concatenated prior to being input in the LSTM. If the LSTM receives observations corresponding to times *T* ∈ {1, 2, 3, …*t* − 1}, then the output of the hidden state will correspond to times *T* ∈ {2, 3, 4, …*t*}. In addition to the predictions, the LSTM outputs the complete hidden state which describes the current state of all elements of the model. The complete hidden state is used during data synthesis as a way of accounting for historical events.

The output layers are a set of linear transformations that take as input the concatenation of the output of the LSTM and the embedded baseline characteristics. These output layers make the predictions for the next time points generated by the LSTM conditioned on the baseline characteristics.

#### Model training

During training, loss is calculated for both the event labels and the event attributes, with masking applied to the event attributes so that only attributes measured for the true event label contribute to the loss. This makes training more efficient as masking the loss for the event attributes restricts the model to learn how to predict each attribute only when it is measured for a given event label.

The event label loss is calculated using cross entropy loss between the predicted event labels and the true event labels:

$$los{s}_{labels}=\frac{1}{Nt}\sum_{n=1}^N\sum_{t=1}^t-{xlabel}_{n,t}\left[{true}_{n,t}\right]+\log \left(\sum_{j=0}^C\exp \left({xlabel}_{n,t}\left[j\right]\right)\ \right)$$

where *xlabel*_*n*, *t*_ is the vector of predicted probabilities for the event label for individual *n* at time *t*, and where *xlabel*_*n*, *t*_[*j*] is the predicted probability that individual *n* at time *t* has event with label *j*, and *true*_*n*, *t*_ is the true event label for individual *n* at time *t*.

Next, cross entropy loss is calculated for the attributes associated with the true event label. For example, if the next time point is truly a lab test, then the model loss for the event attributes is the sum of the cross entropy between the real lab test name and the predicted lab test name and the cross entropy between the real lab test result and the predicted lab test result. This masked form of loss for the event attributes is desirable as it allows the model to focus on learning the relevant features at each time point, rather than constantly predicting missing values.

If we define the indicator function 1(*A*_*i*_ | *true*_*n*, *t*_) to check whether a given attribute *A*_*i*_, is relevant for a given true event label *true*_*n*, *t*_, then cross entropy loss for the attributes is calculated as:

$$los{s}_{attributes}= mean\left\{\sum_{n=1}^N \sum_{t=1}^t \sum_{i=1}^A 1\left({A}_i\ |\ {true}_{n,t}\right)\left[-{x}_{n,t,i}\left[{ true A}_{i,n,t}\right]+\log \left(\sum_{j=0}^C\exp \left({x}_{n,t,i}\left[j\right]\right)\ \right)\right]\right\}$$

where *trueA*_*i*, *n*, *t*_ is the true value for individual *n*’s attribute *i* at time *t* and *x*_*n*, *t*, *i*_ is the vector of the predicted probabilities for individual *n*’s attribute *i* at time *t* among the *C* possible classes for attribute *i*.

Thus, the objective function for training is to minimize the total loss over the model parameters θ, where the tradeoff parameter *λ* controls the relative importance of label loss and attribute loss:

$$\underset{\theta }{\min}\left\{ los{s}_{labels}+\lambda\ los{s}_{attributes}\right\}$$

During training, data is provided for the model in tensors of 120 time points. Individuals have their data grouped into chunks of up to 120 sequential events with 0 s introduced to pad chunks shorter than 120 observations. This is desirable as it produces data that is uniform and much less sparse than if we were to pad up to the true maximum number of observations per individual of 1000.

Hyperparameter optimization was performed using a training set of 100,000 individuals and a validation set of 20,000 individuals. Hyperparameters explored include batch size, number of training epochs, optimization algorithm, learning rate, number of layers within the LSTM, hidden size of the LSTM, embedding size for the event labels, event attributes, and baseline characteristics, and weighting for the different event types and event attributes during calculation of the training loss. Training was performed on an Nvidia P4000 graphics card and was coordinated using Ray Tune.

### Synthetic data generation

After training the model as described in the previous section, synthetic data generation consists of two phases: generation of baseline characteristics and starting values, followed by the generation of longitudinal event data. Baseline characteristics and values for the first event observed are generated using a sequential tree-based synthesis method [88, 89]. Using a scheme similar to sequential imputation [90, 91], trees are used quite extensively for the synthesis of health and social sciences data [52–59, 92]. With these types of models, a variable is synthesized by using the values earlier in the sequence of characteristics as predictors.

These synthesized values are then fed into the trained model to generate the remaining events for each synthetic individual. The goal behind using sequential tree-based synthetic values as the baseline characteristics and starting values for the LSTM model is that they will better reproduce the characteristics of the real population than randomly sampled starting values.

To generate the longitudinal event data, the output of the sequential tree-based synthesis is iteratively fed into the LSTM model. At each iteration, the model uses the synthetic data from the previous time point, as well as the hidden state of the model if available, to predict the next time point. These predictions consisted of predicted event labels and event attributes. Based on the predicted event label, all non-relevant event attributes are masked and set to missing. For example, if the next time point predicts an event of lab tests, the lab test name, lab test result, and sojourn time event attributes will be retained while all others are set to missing. This masking during data generation is important to ensure that the data the model sees during data generation matches the format of the data seen during training. Data synthesis proceeds in this iterative fashion until the model has generated event data up to the maximum sequence length. In post-processing, each sequence is trimmed such that, if available, sequences terminate when a ‘last observation’ event type is observed.

### Generic utility assessments

Generic utility assessments aim to evaluate the similarity between a real and synthetic dataset without any specific use case or analysis in mind. Two types of methods were used depending on whether we were evaluating the utility of the cross-sectional vs the longitudinal portion of the data. All generic utility assessments were completed using python version 3.8.

#### Event distribution comparisons

The simplest generic utility assessments are to compare the number and distribution of events generated for each synthetic individual to the number and distribution of events in the real data. To compare the number of events per individual, the distributions are plotted as histograms and the means are compared. To compare the distribution of events in the real and synthetic data, the observed probability distribution for event types is calculated for each dataset. This corresponds to what proportion of events belongs to each event type. These probability distributions are then plotted and compared as bar charts.

Additionally, these distributions are compared by calculating the Hellinger distance between the two distributions [93]. Hellinger distance is an interpretable metric for assessing the similarity of probability distributions that is bounded between 0 and 1 where 0 corresponds to no difference.

#### Comparing the distribution of event attributes

Another simple metric for assessing the similarity between the real and synthetic datasets is to compare the marginal distributions of each event attribute. For this assessment, we apply the Hellinger distance (as defined above) to the discrete probability distributions for each event attribute. For this assessment, careful consideration is taken to tabulate the probability distributions for each event attribute, only using observations with an event label that is relevant for that attribute. This ensures that we are comparing the distributions of each attribute without the padded/missing values. To summarize the Hellinger distance values calculated for each event attribute, they are plotted in a bar chart.

#### Comparison of transition matrices

The next method we applied for the utility evaluation of synthetic data is to compute the similarity between the real data and the synthetic data transition matrices. A transition matrix reflects the probability of transitioning from one event to another. These transition probabilities can be estimated empirically by looking at the proportion of times that a particular event follows another one.

For example, consider sequence data with four events: A, B, C, and D where C is a terminal event, meaning that if C occurs, a sequence terminates. If 40% of the time an event B follows an event A, then we can say that the transition from A to B has a probability of 0.4. The transition matrix is the complete set of these transition probabilities. Creating such a transition matrix assumes that the next event observed is dependent on only one previous event. This can be quite limiting and does not account for longer term relationships in the data. However, transition matrices can be extended to the *k*^*th*^ order where *k* corresponds to the number of previous events considered when calculating the transition probabilities.

An example of a 2^*nd*^ order transition matrix is shown in Table 3. Here we have the two previous events along with the transition probabilities. The rows indicate the previous states, and the columns indicate the next state. Note that each row needs to add up to 1 because the sum of the total transitions from a pair of consecutive states must be 1. Also, there are no previous states with a C event in them because in our example that is a terminal event.

**Table 3:** An example of a transition matrix with an order of 2, which means that the two previous events are considered. We assume that C is a terminal event

	A	B	C	D
**AB**	0.31	0.29	0.39	0.00
**BA**	0.42	0.21	0.22	0.16
**AD**	0.64	0.11	0.08	0.18
**DA**	0.38	0.05	0.23	0.34
**BD**	0.41	0.31	0.26	0.02
**DB**	0.01	0.16	0.57	0.26
**AA**	0.20	0.40	0.30	0.10
**BB**	0.36	0.34	0.25	0.04
**DD**	0.34	0.48	0.17	0.01

The transition matrices for the real and synthetic datasets can be compared by calculating the Hellinger distance between each row in the real transition matrix and the corresponding row in the synthetic transition matrix. The lower the Hellinger distance values, the closer the transition structure between the two datasets. In this work we report utility for both the 1^*st*^ and 2^*nd*^ order transition matrices.

#### Multivariate Hellinger distance

A multivariate Hellinger distance can be derived from the multivariate normal Bhattacharyya distance [94]. This metric is bound between 0 and 1 and hence is an easily interpreted generic measure of overall similarity of the multivariate distribution between the real and synthetic datasets. This metric has also been shown to be highly predictive of synthetic data utility for logistic regression analyses [95].

#### Utility of random cohorts

All the utility assessments described thus far are conducted on the whole dataset. However, when analyzing longitudinal data, it is quite common to generate queried data (i.e., a cohort) from the whole dataset. Therefore, it is beneficial to compare the cohorts generated by queries on the real and synthetic datasets. A query defines the inclusion and exclusion criteria for the cohort.

For this assessment, we used a fuzzy SQL method. This will generate a large number of random semantically and syntactically correct SELECT random queries that are simultaneously applied to both real and synthetic datasets. The similarity between the resultant real and synthetic cohorts are compared using the Hellinger distance for distributions and normalized Euclidean distance for aggregate results (e.g., the average of a continuous variable in the cohort).

Such SQL fuzzers are used to test database management systems (DBMSs) for any bugs or vulnerabilities [96]. In our context we apply a similar concept to generate random cohorts. More details about our implementation are included in Additional file 1: Appendix 2: Random Cohort Utility Assessment.

### Analysis specific utility assessments

Generic utility assessments are agnostic to the future analyses of the synthetic data and compare the real and synthetic datasets in terms of distributional and structural similarity. In contrast, workload aware or analysis-specific utility assessments compare the real and synthetic datasets by performing the same substantive analysis to both and comparing the results.

For this dataset we also conducted an analysis-specific utility assessment by applying a time-to-event analyses on both the real and synthetic datasets and compared the results.

Our primary outcome was a composite endpoint of all-cause emergency department visits, hospitalization, or death during the follow-up. The secondary outcomes included each component of the composite endpoint separately, as well as to evaluate cause specific admissions to hospital for pneumonia (ICD code J18) as a prototypical example of a cause specific endpoint.

First, all variables in both the synthetic and real data were compared using standard descriptive statistics (e.g., means, medians). Second, standardized mean differences (SMD) were used to statistically compare our variables of interest between the synthetic and real data. SMD was selected as given our large sample size, small clinically unimportant differences, are likely to be statistically different when using t-tests or chi squared test. A SMD greater than 0.1 is deemed as a potentially clinically important difference, a threshold often recommended for declaring imbalance in pharmacoepidemiologic research [97].

Using Cox proportional hazards regression models, unadjusted and adjusted hazard ratios (HRs) and 95% confidence intervals were calculated to assess the risk associated with either morphine or oxycodone and our outcomes of interest in both the synthetic and real data separately. Start of follow-up began on the date of the first dispensation for either morphine or oxycodone. All subjects were prospectively followed until outcome of interest or censoring defined as the date of termination of Alberta Health coverage or 31 March 2018, providing a maximum follow-up of 7 years. Finally, the estimates derived from the real and synthetic datasets were directly statistically compared. Morphine served as the reference group for all estimates. Potential confounding variables included in all multivariate models included age, sex, Elixhauser comorbidity score, use of antidepressant medications, and our 3 laboratory variables (ALT, eGFR, HCT). To compare the confidence intervals estimated for HRs from real vs synthetic dataset, confidence interval overlap was used [98]. All analyses were performed using STATA/MP 15.1 (StataCorp., College Station, TX).

### Privacy assessment

To quantify the privacy risks in the synthetic data we evaluated attribution disclosure risk [42]. This privacy assessment is designed to evaluate the risk that an adversary could match a synthetic with a real record, and that if a re-identification were to occur, whether the adversary would learn something new about them. The quasi-identifiers used for this assessment were: age, sex, death indicator, and a hospitalization indicator. For this assessment, we consider two directions of attack: a population to sample and a sample to population attack [99].

We use the common threshold for the disclosure of clinical trial and other types of health data, 0.09 [100–106], that is the threshold used by the European Medicines Agency for their Policy 0070 anonymization guidance [107], and for Health Canada’s Public Release of Clinical Information guidance [108]. This is equivalent to a minimal group size of 11 under a maximum risk scenario [99].

## Results

### Model parameters

Hyperparameter training was conducted for a variety of aspects of model implementation. By selecting the values within a search range that minimized validation loss, an optimal model was selected. The complete set of optimal values for the hyperparameter can be found in the Additional file 1: Appendix 3: Optimal Model Parameters.

### Generic utility assessments

The generic utility results are summarized in Table 4. They are reviewed in more detail below.

**Table 4:** Summary of the generic utility assessments results

Metric	Result
Percent difference in sequence lengths	0.4%
Hellinger distance of event distribution	0.027
Hellinger distance of event attributes	
Mean (SD)	0.0417
Median (IQR)	0.0303 (0.0333)
Hellinger distance of Markov Transition Matrices of Order 1:	
Mean (SD)	0.0896 (0.159)
Median (IQR)	0.0209 (0.0303)
Hellinger distance of Markov Transition Matrices of Order 2:	
Mean (SD)	0.2195 (0.2724)
Median (IQR)	0.0597 (0.4401)
Multivariate Hellinger distance	0.352
Utility of 100 random cohorts:	
Hellinger distance:	
Mean (SD)	0.3039 (0.0674)
Median (IQR)	0.3128 (0.0358)
Normalized Euclidean distance:	
Mean (SD)	0.0639 (0.1145)
Median (IQR)	0.0146 (0.0596)

**Fig. 3:**
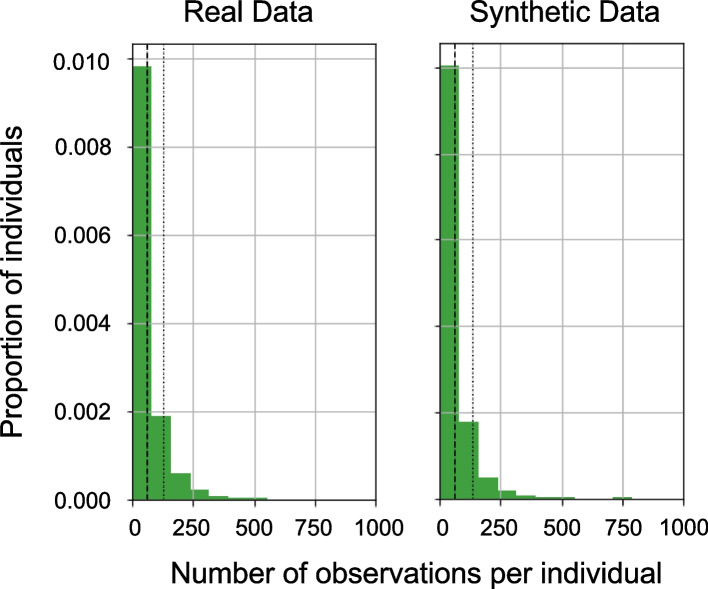
Sequence length comparison between the real and synthetic datasets. Overall, the synthetic data has a similar distribution of sequence lengths than in the real data (real mean & SD: 58.14, 68.57 vs synthetic mean & SD: 58.39, 75.16)

**Fig. 4:**
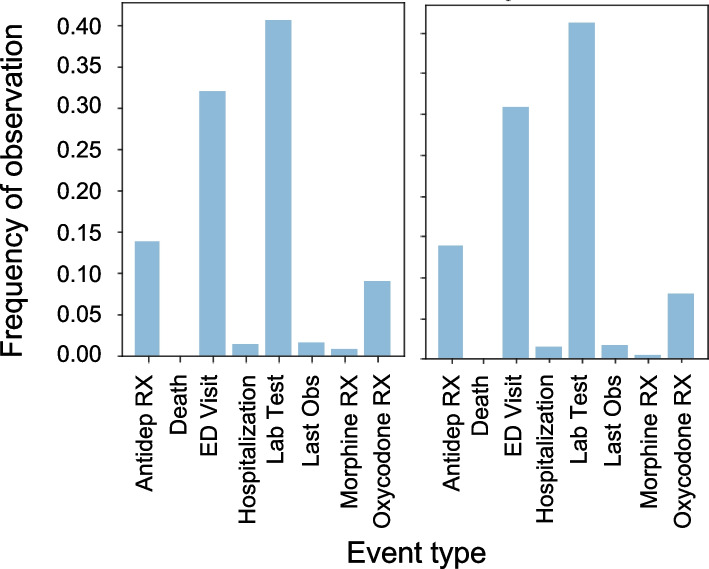
Event distribution comparison between the real and synthetic datasets

The sequence lengths in the synthetic datasets matched the real dataset quite closely (percent difference in mean sequence length 0.4%) as illustrated in Fig. 3. The distribution of events observed across all synthetic patients matched the distribution of events in the real dataset quite closely (Hellinger distance 0.027) as illustrated in Fig. 4.

**Fig. 5:**
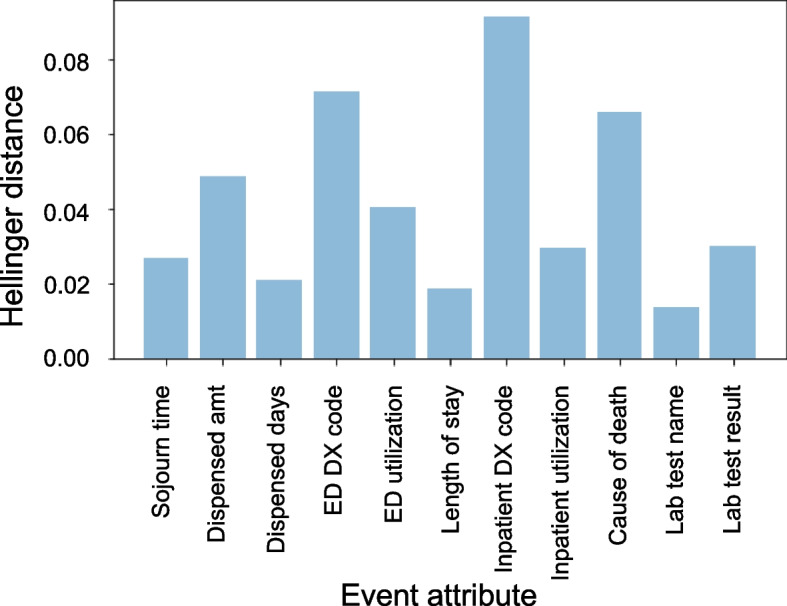
Hellinger distance for each event attribute

**Fig. 6:**
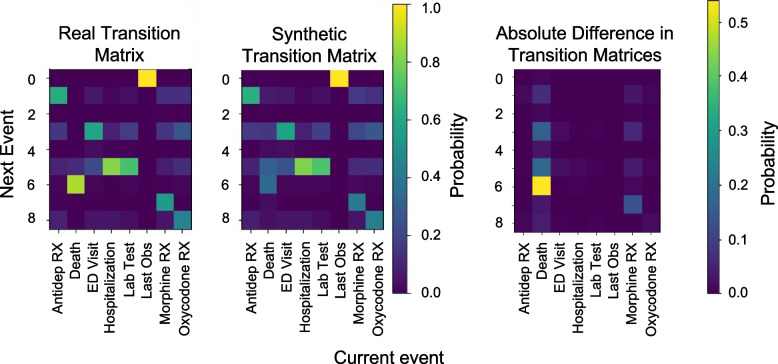
First order Markov transition matrices for the real and synthetic datasets and the absolute difference in transition matrices. Note that the heatmaps have different scales

Comparing the distribution of event attributes, the synthetic data again matches the distributions seen in the real data closely as shown in the Hellinger distance histogram in Fig. 5 (mean Hellinger distance 0.0417). The differences in the real and synthetic transition matrices was smaller for first order Markov transition matrices (in Fig. 6) than for second order transition matrices, (mean Hellinger distance 0.0896 vs 0.2195) indicating that short term dependencies may be modelled better than long term dependencies. The multivariate Hellinger distance shows a distance of 0.352 between the real and synthetic datasets, indicating that the multivariate distributions are moderately similar. The random cohort utility assessment showed a mean Hellinger distance across 100 random cohorts of 0.3039 (standard deviation: 0.0674), and a mean normalized Euclidean distance of 0.0639 (standard deviation: 0.1145). This indicates that randomly generated sub cohorts in the real and synthetic datasets are quite similar. The multivariate Hellinger distance and the random cohort Hellinger results are also similar to each other demonstrating some consistency across utility evaluations.

### Workload aware assessment

The workload aware assessment of utility was conducted on 75,660 real patient records and 75,660 synthetic records. Standardized mean differences (SMD) indicated that no clinically important differences were noted with respect to demographics and the comorbidity score between the real and synthetic data (Table 5). For example, between the real and synthetic data the mean age was 43.32 vs 44.79 (SMD 0.078), 51.0% males vs 52.5% (SMD 0.029), and Elixhauser comorbidity score of 0.96 vs 1.05 (SMD 0.055). However, differences were noted that would be considered potentially clinically important for laboratory data with standardized mean differences between the real and synthetic data > 0.1, a threshold often recommended for declaring imbalance.

**Table 5:** Comparison of trial characteristics across the real and synthetic datasets

	Real ***n*** = 75,660	Synthetic ***n*** = 75,660	SMD
Age			0.078
Mean (SD)	43.32 (17.87)	44.79 (19.83)	
Median (IQR)	42.00 [27.00]	43.00 [30.00]	
Sex n (%)			0.029
Male	38,623 (51.0)	39,711 (52.5)	
Female	37,037 (49.0)	35,949 (47.5)	
Elixhauser			0.055
Mean (SD)	0.96 (1.58)	1.05 (1.63)	
Median (IQR)	0.00 [1.00]	0.00 [2.00]	
ALT			0.099
Mean (SD)	31.67 (63.90)	40.72 (111.92)	
Median (IQR)	24.00 [18.00]	26.00 [19.00]	
eGFR			0.112
Mean (SD)	85.82 (23.56)	83.11 (25.05)	
Median (IQR)	87.00 [41.00]	84.00 [38.00]	
HCT			0.291
Mean (SD)	0.42 (0.05)	0.41 (0.06)	
Median (IQR)	0.42 [0.05]	0.41 [0.06]	
CACS-RIW			0.002
Mean (SD)	0.05 (0.07)	0.05 (0.07)	
Median (IQR)	0.03 [0.03]	0.03 [0.03]	
RIW			0.002
Mean (SD)	1.40 (2.73)	1.40 (2.40)	
Median (IQR)	0.77 [0.82]	0.81 [0.84]	
Opioid Utilization (%)			
Morphine	1758 (2.3)	2649 (3.5)	0.070
Oxycodone	73,902 (97.7)	73,011 (96.5)	
Antidepressant Use	28,224 (37.3)	29,651 (39.2)	0.039

The cumulative follow-up time, post-receipt of the index opioid prescription and the outcomes of interest for the real and synthetic data are summarized in Table 6. Based on SMD cumulative follow-up time (mean of 1474.48 vs 1077.88; SMD: 0.530) and mortality (3299 vs 1440; SMD: 0.141) yielded a notable difference between the real and synthetic datasets.

**Table 6:** Outcomes of interest for both real and synthetic datasets

	Real ***N*** = 75,660	Synthetic ***N*** = 75,660	SMD
Total follow-up time			
Mean (SD)	1474.48 (772.23)	1077.88 (722.44)	0.530
Mortality			
n (%)	3299 (4.4)	1440 (1.9)	0.141
Hospitalization			
n (%)	22,495 (29.7)	21,582 (28.5)	0.027
Emergency room visit			
n (%)	64,376 (85.1)	65,193 (86.2)	0.031
Composite endpoint			
n (%)	64,848 (85.7)	65,497 (86.6)	0.025
Diagnosis of pneumonia (ICD10: J189)			
n (%)	505 (2.2)	472 (2.2)	0.004

**Fig. 7:**
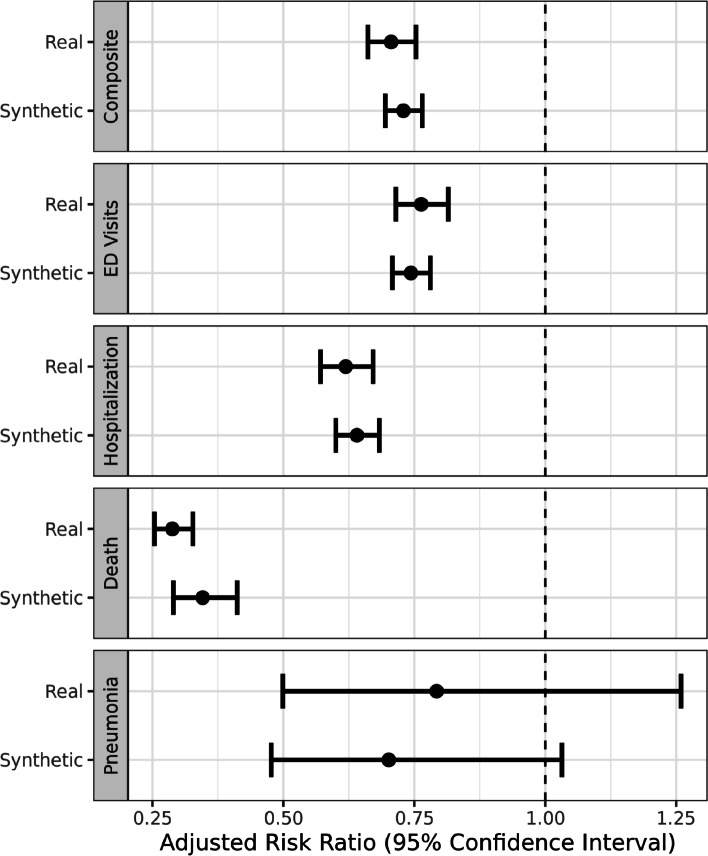
Adjusted hazard ratios for outcomes of interest in the synthetic data compared to the real data

After adjustment for age, sex, use of antidepressants, and laboratory data, the Cox proportional hazards were similar between the real and synthetic datasets. In the real data, oxycodone was associated with a 29% reduction in time to composite endpoint compared to morphine: adjusted HR (aHR) 0.71 95% CI 0.66–0.75). A similar reduction was observed in the synthetic dataset with a 27% reduction in time to event: aHR 0.73 95% CI 0.69–0.77 (Fig. 7 and Table 7). With respect to our secondary outcomes, similar trends were observed with small differences noted in time to event between the synthetic and real data with the exception of all-cause mortality (Fig. 7). With respect to all-cause mortality, although both the real and synthetic data would provide similar conclusions on the effect of oxycodone on mortality, the estimated effect was higher in the real data, with only a 38% confidence interval overlap (aHR 0.29 (95% CI 0.25, 0.33) vs aHR 0.35 (95% CI 0.29, 0.41)).

**Table 7:** Adjusted hazard ratios and confidence interval overlap for outcomes of interest in real and synthetic datasets

Outcome	Real Data	Synthetic Data	CI-Overlap-percent
Mortality	0.29 (0.25, 0.33)	0.35 (0.29, 0.41)	38%
Hospitalization	0.62 (0.57, 0.67)	0.64 (0.6, 0.68)	77%
Emergency room visit	0.76 (0.71, 0.81)	0.74 (0.71, 0.78)	76%
Composite endpoint	0.71 (0.66, 0.75)	0.73 (0.69, 0.77)	72%
Pneumonia	0.79 (0.5, 1.26)	0.7 (0.48, 1.03)	81%

The confidence intervals and point estimates in the adjusted Cox regression analysis are also similar and would lead researchers to reach the same conclusion for many applications whether they analyzed real or synthetic datasets. For the adjusted models the mean confidence interval overlap is 68%. This indicates that the conclusions drawn from the synthetic datasets comfortably overlap those drawn from the real data.

### Privacy assessment results

The privacy assessment showed that the population to sample risk was 0.001476 and the sample to population risk was 0.001474. Given that both these risk values are substantially lower than the acceptable risk threshold of 0.09, we can conclude that the attribution disclosure risks associated with this synthetic dataset is acceptably low.

## Discussion and conclusions

### Summary

This project has generated realistic synthetic data for complex longitudinal administrative health records. Modelling events over time using a form of conditional LSTM has allowed us to learn patterns in the data over time, as well as how these trends relate to fixed baseline characteristics. The masking implemented during model training has allowed us to work with sparse attribute data from a variety of sources in a single model. Overall, this method of generating synthetic longitudinal health data has performed quite well from a data utility perspective.

Generic univariate and multivariate utility metrics based on the Hellinger distance varied from a low of 0.01 for event attributes, to 0.35 for the joint distributions. Random cohort generation also had a mean Hellinger distance of 0.3 between real and synthetic cohorts generated from longitudinal data.

Our model learns and recreates patterns in the heterogeneous attributes, accounting for the pattern of relevant attributes based on event type. The generated sequences have event lengths that are consistent with the real data (percent difference in mean sequence length 0.4%). Baseline characteristics were synthesized to be consistent with the distributions in the real data (SMD 0.05 or lower) and to exert reasonable influence on the progression of events. There were differences in the univariate lab results between the real and synthetic datasets.

The multivariate Cox models incorporating the main variables of interest and confounders used to predict multiple outcomes were similar between real and synthetic data, with confidence interval substantial overlap on the effect of Oxycodone (mean CI overlap above 68%). Our work has shown the ability of synthetic data to reproduce results of traditional epidemiologic analyses reasonably well. Additionally, we have demonstrated in this study that the privacy risks associated with this synthetic dataset are acceptably low when considering population to sample attacks (estimated risk: 0.001476) and sample to population attacks (estimated risk: 0.001474).

### Contributions of this work

The conditional LSTM generative model described in this paper has worked well with real-world complex longitudinal data that has received minimal curation. This method allows the synthesis of associated cross sectional and longitudinal health data, where the measures included correspond to a variety of medical events (e.g., prescriptions, doctor visits, etc.) and data types (e.g., continuous, binary, categorical). The longitudinal data generated varies in the number of observations per individual, reflecting the structure of real electronic health data. The model selected is easy to train and automatically adapts as the number of events, event attributes, or complexity of attributes changes.

We have also assessed the utility of the generated synthetic data using generic and workload aware assessments that have shown the similarity of our generated data to the real data on most univariate measures and for multivariate models. The privacy assessment has shown that the risks from the synthetic data generated are below generally accepted risk thresholds.

Architecturally, the generative model has a number of features which make it suitable for this type of data:

- Combining a tabular generative model as an input to the longitudinal generative model.
- Using masking on the loss function to focus only on the relevant attributes at a particular point in time.
- Dynamically weighting the loss for event attributes and event labels.
- The multiple embedding layers allow the model to handle heterogeneous data types.

The above features enabled the model to learn the patterns in the original dataset.

### Limitations and future work

Our methods truncated the maximum sequence length at the 95th percentile. This means that data from individuals with the greatest number of interactions with the healthcare system are not modelled nor synthesized, and therefore our synthetic data may not be applicable to those interested in assessing the impacts of high healthcare utilization individuals.

This generative model was designed to learn and reproduce the relationships seen in the training dataset without tuning or optimizing for a specific analysis. Higher utility results may be achieved by tuning a synthesis model to a specific analysis, however that may come at the cost of the generalizability of the data generated.

The approach we used in this study to compare the confidence intervals between the real and synthetic datasets did not account for the additional variance introduced by synthesis. While combining rules similar to those used for multiple imputation can be used to account for the additional variance [109, 110], some authors have suggested that parameter estimates and confidence intervals computed from a single synthetic dataset can still be valid [57]. Future work should examine the additional benefit of considering this multiple imputation approach.

While there is a body of work on the synthesis of medical images and other data types [111], our focus in this paper was on structured longitudinal data. The synthesis of multi-modal data would be an important direction for future research.

## Supplementary Information

**Additional file 1: Appendix 1.** Data Pre-Processing. **Appendix 2.** Random Cohort Utility Assessment. **Appendix 3.** Optimal Model Parameters.

## Data Availability

The data that support the findings of this study were obatined from Alberta Health Services but restrictions apply to the availability of these data, which were used under license for the current study, and so are not publicly available. The dataset can be requested from Alberta Health Services under their data sharing program: https://www.albertahealthservices.ca/research/Page16074.aspx. An illustrative example of the analysis code is available from the authors upon request. Code to run the utility of random cohorts fuzzy SQL assessment are available here: https://github.com/skababji-ehil/fuzzy_sql.
