# Supplementary material for: A method for generating synthetic longitudinal health data
Source: BMC Med Res Methodol. 2023 Mar 23;23:67. doi: 10.1186/s12874-023-01869-w (PMC10034254; doi:10.1186/s12874-023-01869-w)
Supplement: Supplementary file 1 — Additional file 1: Appendix 1. Data Pre-Processing. Appendix 2. Random Cohort Utility Assessment. Appendix 3. Optimal Model Parameters. [file 12874_2023_1869_MOESM1_ESM.pdf]

# Supplementary Materials

## 1. Appendix 1: Data Pre-Processing

The main steps of data pre-processing can be grouped as modifying the data structure and variable encoding. The goal of modifying the data structure is combining the different original data tables into a format that is suitable for the RNN. In contrast, variable encoding aims to format each variable in the dataset in a manner that is suitable for the RNN.

### 1.1 Modifying Data Structure

The original structure of the data provided consisted of multiple forms linked by a single subject identifier where each form consists of a single type of health information. The goal of modifying the data structure is to transform these tables into a consistent representation for our machine learning model. We group data based on whether they are longitudinal events that occur over time, compared to baseline characteristics.

In this dataset the baseline characteristics include the age, sex, and baseline comorbidity index for the individual. Additionally, the relative date of the individual's first observation is included as a baseline characteristic. These measures are then combined in a single dataset $BC$ that has the structure in Table 1.

$BC = [n, B]$

| Encrypted PHN | Age | Sex | Comorbidity | Relative Date of First Obs |
|---|---|---|---|---|
| 10000001 | 38 | F | 0 | 100 |
| 10000002 | 22 | M | 0 | 325 |
| 10000003 | 70 | F | 1 | 52 |
| 10000004 | 55 | F | 0 | 89 |
| 10000005 | 63 | M | 3 | 600 |

**Table 1:** Structure of baseline characteristic (BC) data. This produces a table of size $BC = [n, B]$, where $n$ corresponds to the number of individuals in the dataset and $B$ corresponds to the number of baseline characteristics present in the data. In this case $B = 4$.

Longitudinal events include prescriptions, physician visits, hospitalizations, emergency department visits, and. We joined these observations from different data tables by assigning event type labels and associated attributes for each event type. For example, all observations from the hospitalization form are considered the event 'hospitalization' and have measures for the attributes: length of stay and resource intensity

weight. Given that not every attribute is measured for every event type, this yields a sparse data frame with many missing values for event attributes. Table 2 illustrates the structure of the joined data frame. This data frame captures all events that occur throughout the study period for each patient.

All original data tables correspond to a single event type (e.g., the hospitalization form yield 'hospitalization' events), except for the drug_data and MD claims forms. These two forms have 47 million and 29 million observations respectively, which constitutes 83% of the total number of event observations. To prevent strong imbalance between different event types, the drug_data form was split into 4 event types: morphine dispensations, oxycodone dispensations, antidepressant dispensations, and other prescription dispensations while the MD claims form was split into 2 event types: general practitioner visits and specialist visits. This split leverages the existing features in the data.

After joining observations from the different transactional tables, relative dates for each event were recoded as time between events or sojourn time. This transformation was conducted as longitudinal health data is often utilized for time to events type analyses, and therefore we prioritized modelling the time between events rather than the relative dates of observations.

One important characteristic of this dataset is the wide range in the number of observations associated with each individual. Summarized as percentiles in Table 3, we can see that most patients have dozens or hundreds of events recorded, while very few (<5% of patients) have between 1,000 and 36,774 events recorded. This great range in number of events is something we are interested in preserving, that also may be associated with the features of the data itself (i.e. individuals with more observations may be sicker so they are more likely to have ongoing prescriptions, chronic conditions, etc.). For simplicity, patients with >1000 observations were omitted from the dataset, which is a cut at the 95$^{th}$ percentile of event counts as shown in Table 3. This still provides a considerable amount of variability in the number of events.

| Encrypted PHN | Label | Sojourn Time | Amt Dispensed | Duration of RX | ICD10 Diagnostic Code | RIW | LOS | Specialist Type | ICD9 Diagnostic Code | Lab Test Name | Lab Test Results |
|---|---|---|---|---|---|---|---|---|---|---|---|
| 1000001 | GP Visit | 0 | NA | NA | NA | NA | NA | NA | 311 | NA | NA |
| 1000001 | Other RX | 0 | 10 | 7 | NA | NA | NA | NA | NA | NA | NA |
| 1000001 | Antidep RX | 0 | 100 | 60 | NA | NA | NA | NA | NA | NA | NA |
| 1000001 | MD Visit | 62 | NA | NA | NA | NA | NA | ORTH | 724.5 | NA | NA |
| 1000001 | Morphine RX | 0 | 30 | 60 | NA | NA | NA | NA | NA | NA | NA |
| 1000001 | Lab Test | 2 | NA | NA | NA | NA | NA | NA | NA | GFR | 85 |
| 1000001 | GPVisit | 180 | NA | NA | NA | NA | NA | NA | 724.5 | NA | NA |
| 1000001 | Morphine RX | 0 | 60 | 60 | NA | NA | NA | NA | NA | NA | NA |
| 1000001 | ED Visit | 5 | NA | NA | N20.0 | 0.001 | NA | NA | NA | NA | NA |
| 1000001 | Hospitalization | 10 | NA | NA | I75.81 | 0.05 | 7 | NA | NA | NA | NA |
| 1000001 | Oxycodone RX | 0 | 120 | 7 | NA | NA | NA | NA | NA | NA | NA |
| 1000001 | Death | 7 | NA | NA | I75.81 | NA | NA | NA | NA | NA | NA |
| 1000001 | Last Obs | 0 | NA | NA | NA | NA | NA | NA | NA | NA | NA |

**Table 2:** Structure of joined longitudinal dataset. This snapshot shows the structure of data for a single patient up to their death. NA values indicate not applicable or missing values.

| Percentile | 0% | 5% | 10% | 15% | 20% | 25% | 30% | 35% | 40% | 45% | 50% |
|---|---|---|---|---|---|---|---|---|---|---|---|
| # Obs | 2 | 25 | 40 | 54 | 69 | 84 | 99 | 116 | 134 | 153 | 175 |
| **Percentile** | **55%** | **60%** | **65%** | **70%** | **75%** | **80%** | **85%** | **90%** | **95%** | **100%** | |
| # Obs | 199 | 227 | 260 | 299 | 349 | 414 | 507 | 660 | 997 | 36774 | |

**Table 3:** Percentiles for the number of events per patient.

## 1.2 Variable Encoding

For the formatted datasets described in Table 1 and Table 2 to be suitable for the RNN, feature encoding must occur. Feature encoding helps ensure that all features the model is attempting to learn are on similar scales. When minimizing error in prediction, features with larger ranges and thus larger prediction errors will be prioritized during training. This is not a desirable trait as we would like each feature to be prioritized equally unless we specify otherwise. For the LSTM models we are applying, in order to make the training process easier, all features are discretized.

The kind of feature encoding performed depends on the format of the original variable. In this dataset the following transformation were performed:

- Categorical variables with ≤100 levels: (e.g., lab test name, specialist type, event labels) were mapped 1 to 1 from the text categories to the integers 1, 2, 3, etc.
- Continuous variables: (e.g., sojourn time, dispensed amount, prescription duration, length of stay, resource intensity weight, lab test result) were binned and then mapped to the integers 1, 2, 3, etc.
- Categorical variables with >100 levels: (e.g., ICD9 and ICD10 diagnostic codes) were formatted based on prevalence in the data. Levels with many observations were kept in their original format, while levels that were less common were generalized to the chapter level.
- Baseline characteristics: were left in their original format, except for date of first observation. Date of first observation was scaled based on the study period (i.e., if the first observation for an individual was recorded on day 200, this was transformed using the 7 year, or 2557 days, study period to be $\frac{200}{2557} = 0.078$).

# 2. Appendix 2: Random Cohort Utility Assessment

In this appendix, we describe the random cohort utility assessment or fuzzy SQL tool in detail. The data types handled by the fuzzy SQL tool are shown in 2.1. Then more details about the generated queries are given in 2.2 and the utility metrics can be found in 2.3.

## 2.1 The data types

To ensure the validity of the SQL select statement as interpreted by the database engine, Fuzzy SQL makes a distinction among three basic data types, namely: Categorical, Continuous, and Date. Accordingly, if a dataset includes a variable with a different data type, it will be mapped to the proper type as per the table below:

| Input Data Type | Output Data Type |
|---|---|
| 'qualitative', 'categorical', 'nominal', 'discrete', 'ordinal', 'dichotomous', 'TEXT', 'INTEGER' | 'categorical' |
| 'quantitative', 'continuous', 'interval', 'ratio', 'REAL' | 'continuous' |
| 'date', 'time', 'datetime' | 'date' |

The distinction arises from their intrinsic properties as summarized in the following table:

| Property | 'categorical' | 'continuous' | 'date' |
|---|---|---|---|
| Can be used to aggregate data across it | Yes | No | No |
| Can be used with the aggregate functions: SUM, AVG, MIN and MAX | No | Yes | No |
| Can be used with the 'IN' operation | Yes | No | Yes |
| Can be used with the 'BETWEEN' operation | No | Yes | Yes |

The three basic data types will be equally used for the rest of the query operations.

## 2.2 Data Query Templates

Without loss of generality, and to simplify the mathematical constructs, we herein ignore the logical operation 'NOT' and the value comparison operations BETWEEN, LIKE, and IN. We further consider that the same set of value comparison operations is applicable to all types of variables. In practice, a distinction in their applicability is made and all the aforementioned operations are considered. Further, please note that, for our purpose, the terms *categorical* and *nominal* are used interchangeably. Define:

- $\mathcal{T}^r$ : Database table for real data.
- $\mathcal{T}^s$: Database table for synthetic data
- $N$: The number of records in $\mathcal{T}^r$.
- $\mathbb{A}^n = \{A_1^n, A_2^n, \cdots, A_{|\mathbb{A}^n|}^n\}$ is the set of *nominal* variables in both $\mathcal{T}^r$ and $\mathcal{T}^s$ where $|\mathbb{A}^n|$ indicates the number of these variables.
- $\mathbb{A}^c = \{A_1^c, A_2^c, \cdots, A_{|\mathbb{A}^c|}^c\}$ is the set of *continuous* variables in both $\mathcal{T}^r$ and $\mathcal{T}^s$.
- $\mathbb{A}^d = \{A_1^d, A_2^d, \cdots, A_{|\mathbb{A}^d|}^d\}$ is the set of *date* variables in both $\mathcal{T}^r$ and $\mathcal{T}^s$.
- For any member $A_j$ in the above sets we denote $V(A_j)$ as the set of all values that $A_j$ may take. The length of $V(A_j)$ is $|V(A_j)| \leq N$.

We further define various operations:

- $LO = \{AND, OR\}$ is the set of logical operations.
- $CO = \{=, \neq, <, \leq, >, \geq\}$ is the set of value comparison operations.
- $AG = \{SUM, AVG, MIN, MAX\}$ is the set of aggregate functions.

Random samples are drawn from the above sets to construct the three major queries defined below. The basic sampling functions can be defined as:

$f_s: S_m \rightarrow S_s$ where $f_s$ is a sampling function that maps any set $S_m$ into a single element set $S_s$. For instance, the set $AG$ may be mapped by $f_s$ into $\{AVG\}$

$f_m: S_{m1} \rightarrow S_{m2}$ where $f_m$ is a sampling function that maps any set $S_{m1}$ into a multiple element set $S_{m2}$. For instance, the set $\mathbb{A}^n$ may be mapped by $f_m$ into $\{A_1^n, A_{|\mathbb{A}^n|}^n\}$.

### 2.2.1 Aggregate Queries

If $\mathbb{A}^c = \phi$, an aggregate query takes the form:

SELECT $f_m(\mathbb{A}^n)$, COUNT(*)
FROM $\mathcal{T}^r$
GROUP BY $f_m(\mathbb{A}^n)$

However, if $\mathbb{A}^c \neq \phi$, an aggregate query takes the form:

SELECT $f_m(\mathbb{A}^n), f_s(AG)(f_s(\mathbb{A}^c))$, COUNT(*)
FROM $\mathcal{T}^r$
GROUP BY $f_m(\mathbb{A}^n)$

Similar queries are constructed for $\mathcal{T}^s$.

### 2.2.2 Filter Queries

If $\mathbb{A}^c = \phi$, a filter query takes the form:

SELECT $*$
FROM $\mathcal{T}^r$
WHERE $[f_s(\mathbb{A}^n \cup \mathbb{A}^c \cup \mathbb{A}^d) \quad f_s(CO) \quad f_s(V(f_s(\mathbb{A}^n \cup \mathbb{A}^c \cup \mathbb{A}^d)))]$
$[f_s(LO)]$
$[f_s(\mathbb{A}^n \cup \mathbb{A}^c \cup \mathbb{A}^d) \quad f_s(CO) \quad f_s(V(f_s(\mathbb{A}^n \cup \mathbb{A}^c \cup \mathbb{A}^d)))]$
$[f_s(LO)]$
$\cdots$

The WHERE clause comprises basic expressions denoted by [ ]. Say if the sampled number of variables equals to 1 (i.e. $|f_m(\mathbb{A}^n \cup \mathbb{A}^c \cup \mathbb{A}^d)|$=1), then the above expression will reduce to:

SELECT $*$
FROM $\mathcal{T}^r$
WHERE $(f_s(\mathbb{A}^n \cup \mathbb{A}^c \cup \mathbb{A}^d) \quad f_s(CO) \quad f_s(V(f_s(\mathbb{A}^n \cup \mathbb{A}^c \cup \mathbb{A}^d)))$

If $\mathbb{A}^c \neq \phi$, a filter query takes the form:

SELECT $f_s(AG)(f_s(\mathbb{A}^c))$, COUNT(*)
FROM $\mathcal{T}^r$
WHERE $[f_s(\mathbb{A}^n \cup \mathbb{A}^c \cup \mathbb{A}^d) \quad f_s(CO) \quad f_s(V(f_s(\mathbb{A}^n \cup \mathbb{A}^c \cup \mathbb{A}^d)))]$
$[f_s(LO)]$
$[f_s(\mathbb{A}^n \cup \mathbb{A}^c \cup \mathbb{A}^d) \quad f_s(CO) \quad f_s(V(f_s(\mathbb{A}^n \cup \mathbb{A}^c \cup \mathbb{A}^d)))]$
$[f_s(LO)]$
$\cdots$

Similar queries are constructed for $\mathcal{T}^s$.

### 2.2.3 Filter-Aggregate Queries

Filter-Aggregate queries are the most important for comparing real and synthetic datasets. The query is constructed by combining the above two forms. Hence, if $\mathbb{A}^c = \phi$, a filter-aggregate query takes the form:

SELECT $f_m(\mathbb{A}^n)$, COUNT(*)
FROM $\mathcal{T}^r$
WHERE $[f_s(\mathbb{A}^n \cup \mathbb{A}^c \cup \mathbb{A}^d) \quad f_s(CO) \quad f_s(V(f_s(\mathbb{A}^n \cup \mathbb{A}^c \cup \mathbb{A}^d)))]$
$[f_s(LO)]$
$[f_s(\mathbb{A}^n \cup \mathbb{A}^c \cup \mathbb{A}^d) \quad f_s(CO) \quad f_s(V(f_s(\mathbb{A}^n \cup \mathbb{A}^c \cup \mathbb{A}^d)))]$
$[f_s(LO)]$

$\cdots$

GROUP BY $f_m(\mathbb{A}^n)$

and if $\mathbb{A}^c \neq \phi$, a filter-aggregate query takes the form:

SELECT $f_m(\mathbb{A}^n), f_s(AG)(f_s(\mathbb{A}^c))$, COUNT(*)
FROM $\mathcal{T}^r$
WHERE $[f_s(\mathbb{A}^n \cup \mathbb{A}^c \cup \mathbb{A}^d) \quad f_s(CO) \quad f_s(V(f_s(\mathbb{A}^n \cup \mathbb{A}^c \cup \mathbb{A}^d)))]$
$[f_s(LO)]$
$[f_s(\mathbb{A}^n \cup \mathbb{A}^c \cup \mathbb{A}^d) \quad f_s(CO) \quad f_s(V(f_s(\mathbb{A}^n \cup \mathbb{A}^c \cup \mathbb{A}^d)))]$
$[f_s(LO)]$
$\cdots$
GROUP BY $f_m(\mathbb{A}^n)$

Similar queries are constructed for $\mathcal{T}^s$.

## 2.3 Utility Metrics

In the explanations below we use examples from the Adult datasets available from the UCI machine learning repository.

### 2.3.1 Hellinger Distance for Datasets

The Hellinger distance is used to measure the quality of synthetic data. First, we consider the calculation of the Hellinger distance between the real and the synthetic tabular datasets $\mathcal{T}^r$ and $\mathcal{T}^s$ respectively. Define:

- $\mathbb{A} = \{A_1, \cdots, A_i, \cdots, A_{|\mathbb{A}|}\}$ is the set of *nominal* variables in both $\mathcal{T}^r$ and $\mathcal{T}^s$ where $|\mathbb{A}|$ indicates the number of these variables.
- $o_{A_i}^j$ is the number of occurrences (i.e. counts) of the $j^{th}$ class for the nominal variable $A_i$ in $\mathcal{T}^r$.

The discrete probability of the $j^{th}$ class can be calculated as:

$$r_{A_i}^j = \frac{o_{A_i}^j}{\sum_{\forall j} o_{A_i}^j}$$

For instance, consider the *nominal* variable $A_1 =$ "income" with two classes '<=50k' and '>50k'. Then the first class may have $o_{A_1}^1 = 1200$ occurrences and the second may have $o_{A_1}^2 = 2000$ occurrences with discrete probabilities of $r_{A_1}^1 = 0.375$ and $r_{A_1}^2 = 0.625$ respectively.

Similarly, for the synthetic data $\mathcal{T}^s$, we can calculate the discrete probabilities $s_{A_i}^j$

The Hellinger distance for the nominal variable $A_i$ is calculated as:

$$\mathcal{H}^{A_i} = \frac{1}{\sqrt{2}} \left( \sum_{\forall j} \left( \sqrt{r_{A_i}^j} - \sqrt{s_{A_i}^j} \right)^2 \right)^{1/2}$$

The Hellinger distance between $\mathcal{T}^r$ and $\mathcal{T}^s$ can be calculated by taking the mean across all *nominal* variables:

$$\mathcal{H}^{\mathcal{T}} = \frac{1}{|\mathbb{A}|}\sum_{i=1}^{|\mathbb{A}|}\mathcal{H}^{A_i}$$

### 2.3.2 Hellinger Distance for Cohort

In *aggregate* queries, grouping is done by randomly selected *nominal* variables. In this sense, measuring the Hellinger distance for the datasets as explained above is just a special case where grouping is done by a single nominal variable at a time. So, for $|\mathbb{A}|$ number of *nominal* variables in the original datasets, we may execute $|\mathbb{A}|$ number of queries with each query grouped by a single variable. Then by averaging the Hellinger distances of these queries, we reach the same results in 1.3.1

If grouping is done by more than a single variable, it is as if we are defining a new nominal variable $A^q$ where $A^q$ may be any combination of two or more dataset variables $A_i \quad \forall A_i \in \mathbb{A}$ as defined in 1.3.1. The query will result in a specific number of classes for $A^q$. Using the superscript $j$ to indicate the $j^{th}$ class of $A^q$, we calculate the Hellinger distance for the query by:

$$\mathcal{H}^{\mathcal{Q}} = \frac{1}{\sqrt{2}}\left(\sum_{\forall j}\left(\sqrt{r_{A^q}^j} - \sqrt{s_{A^q}^j}\right)^2\right)^{1/2}$$

Both discrete probabilities $r$ and $s$ were defined earlier in 1.3.1. For instance, consider an aggregate query grouped by the two nominal variables $A_1 =$ "income" and $A_2 =$ "marital status" with each having two distinct classes. The query will result in the variable $A^q$ having four distinct classes with a discrete probability $r_{A^q}^j$ for each resulting class $j$.

### 2.3.3 Euclidean Distance for Cohort

Once the *aggregate* query is executed, the variable $A^q$, as defined in 1.3.2, will result in the classes: $1,2..j..J$. If the data includes a continuous variable $A^c$, an aggregate function, say AGG, may be applied to that variable. For each class $j$, an aggregation value $[AGG(A^c)]_j$ of the continuous variable can be calculated. For instance, let $A^q$ be a combination of two nominal variables $A_1 =$ "income" and $A_2 =$ "marital status". Let $A^c =$ "age" be a continuous variable, then for each of the four distinct classes, we can calculate the AVG(age). Define:

- $v_j^r$ is the aggregate value (e.g., $[\text{AVG(age)}]_j$) corresponding to the $j^{th}$ class of an arbitrary continuous variable $A^c$ in $\mathcal{T}^r$.
- $v_j^s$ is the aggregate value corresponding to the $j^{th}$ class of the same continuous variable $A^c$ in $\mathcal{T}^s$

From the above components, we can find the different components:

$$d_j = v_j^r - v_j^s \quad \forall j$$

We further find the mean and standard deviation across all the classes:

$$\mu^d = \frac{1}{J}\sum_{j=1}^{J} d_j$$

$$\sigma^d = \sqrt{\frac{1}{J}\sum_{j=1}^{J}(d_j - \mu^d)^2}$$

and we compute the standardized aggregate values:

$$z_j = \frac{d_j - \mu^d}{\sigma^d}$$

Finally, we compute the norm and normalize it to reflect the normalized Euclidean distance between the real and synthetic queries:

$$\mathcal{E}^Q = \frac{\| z_j \|}{J}$$

Normalizing the distance by the number of resulting classes for the random query enables us to average the Euclidean distance across multiple queries since each of them may result in different number of classes.

### 2.3.4 Example for calculating distances

As a simple example, consider a real $\mathcal{T}^r$ dataset with the variables $\mathbb{A}^n = \{work\_class, education\}$ and $\mathbb{A}^c = \{hours\_per\_week\}$. A random aggregate query is executed and resulted in the following:

| work_class | education | AVG(hours_per_week) | COUNTS |
|---|---|---|---|
| Private | Divorced | 39.0 | 219 |
| Self-emp | Married | 33.3 | 9 |
| Private | Married | 41.0 | 29 |
| Local-gov | Never-married | 36.0 | 20 |
| Private | Never-married | Nan | Nan |

Aggregate random query applied to $\mathcal{T}^r$

| work_class | education | AVG(hours_per_week) | COUNTS |
|---|---|---|---|
| Private | Divorced | 35.0 | 121 |
| Self-emp | Married | 40.0 | 30 |
| Private | Married | 38.5 | 21 |
| Local-gov | Never-married | Nan | Nan |
| Private | Never-married | 39.0 | 3 |

Aggregate random query applied to $\mathcal{T}^s$

The query results in a new variable $A^q$ which is the combination of $A_1 = work\_class$ and $A_2 = education$. The resulting variable assumes new classes which are basically the combination of the classes of the original variables. Further, the executed random query does not necessarily result in the same *number* and *type* of records (i.e. classes) for both real and synthetic data. This explains the presence of Nan which indicates a non-matching record. This can go both sides, i.e., synthetic data may miss a class combination that is present in the real data, or it may result in a combination that is never present in the real data.

In the tables above, the real data query resulted in 277 occurrences of the first four classes (i.e. (Private, Divorced) with a probability 219/277=0.791, (Self-emp, Married) with a probability of 9/277=0.0325 and so forth. The synthetic data query resulted in a total of 175 occurrences and its class probabilities can be calculated in the same manner.

Replacing Nan by a probability of zero, the Hellinger distance between the two queries is calculated as

$$\mathcal{H}^Q = \frac{1}{\sqrt{2}}((\sqrt{0.791} - \sqrt{0.691})^2 + (\sqrt{0.325} - \sqrt{0.171})^2 + \cdots)^{1/2} = 0.0523$$

For the Euclidean distance, we first find the different components of the resulting averages of the continuous variable hours_per_week as (4, -0.67,2.5). Note that the non-matching classes are ignored. The component vector can be standardized into (0.702,-1.145,0.443) and finally, the norm is calculated and divided by the number of components resulting in a normalized Euclidean distance of 0.471.

# 3. Appendix 3: Optimal Model Parameters

Hyperparameter optimization for generating each of the synthetic datasets. Optimization was performed on a Nvidia p4000 GPU.

| | **Optimal Value** |
|---|---|
| Batch Size | 256 |
| Training Epochs | 50 |
| Learning Rate | $8.98 \times 10^{-6}$ |
| Optimization Algorithm | ADAM |
| LSTM Layers | 1 |
| LSTM Hidden Size | 648 |
| Embedding Size for Baseline Characteristics | [sex: 3, elixhauser: 9, age: 13] |
| Embedding Size for Event Labels | 29 |
| Embedding Size for Event Attributes | [sojourn time: 8, dispensed amount: 12, dispensed days: 12, ED diagnostic code: 18, ED RIW: 12 , hospitalization length of stay: 12, hospitalization diagnostic code: 8, hospitalization RIW: 12, cause of death: 12, lab test name: 9, lab test result: 12] |

**Table 4:** Optimal model parameters as selected via hyperparameter optimization.
